# Last exit before the brink: Conservation genomics of the Cambodian population of the critically endangered southern river terrapin

**DOI:** 10.1002/ece3.5434

**Published:** 2019-08-05

**Authors:** F. Gözde Çilingir, Adeline Seah, Brian D. Horne, Sitha Som, David P. Bickford, Frank E. Rheindt

**Affiliations:** ^1^ Department of Biological Sciences National University of Singapore Singapore City Singapore; ^2^ Wildlife Conservation Society, Bronx Zoo Bronx New York USA; ^3^ Wildlife Conservation Society Phnom Penh Cambodia; ^4^ Present address: Department of Biology University of La Verne La Verne California USA

**Keywords:** Batagur, Cambodia, conservation genetics, ddRAD‐seq

## Abstract

The southern river terrapin, *Batagur affinis* is one of the world's 25 most endangered freshwater turtle species. The major portion of the global population is currently found in peninsular Malaysia, with the only remnant Indochinese population in southern Cambodia. For more than a decade, wild nests in this remnant Cambodian population have been fenced and hatchlings reared in captivity. Here we amplified 10 microsatellite markers from all 136 captive individuals, obtained 2,658 presumably unlinked and neutral single nucleotide polymorphisms from 72 samples with ddRAD‐seq, and amplified 784 bp of mtDNA from 50 samples. Our results reveal that the last Indochinese population comprised only four kinship groups as of 2012, with all offspring sired from <10 individuals in the wild. We demonstrate an obvious decrease in genetic contributions of breeders in the wild from 2006–2012 and identify high‐value breeders instrumental for ex‐situ management of the contemporary genetic stock of the species.

## 1 INTRODUCTION

The impact of genetic factors on a species' extinction risk can be severe. Decreased genetic diversity can be translated into a loss of long‐term evolutionary potential, and inbreeding depression is one of the most pervasive extinction mechanisms reducing reproductive success and survival in populations (Frankham, 2005; Reed & Frankham, 2003). As most endangered species or populations undergo steep declines, the loss of genetic diversity in a severely depleted population has the potential to expedite the extinction process.

As wild populations decrease in size to critical levels, ex‐situ conservation programmes and reintroductions of captive animals have become the most widespread measures to protect endangered species (Primack, 1993). The major goal of assurance colony management (compilation of captive founders) and captive breeding programmes is to produce self‐sustaining captive stock that resembles its bottlenecked wild counterparts as closely as possible both in behavior and genetic profile (Frankham, 2008; Frankham, Briscoe, & Ballou, 2010; Robert, 2009). Consequently, genetic management of captive stock aims for minimizing kinship between potential founders to preserve maximum genetic variability and minimize inbreeding in captive bred individuals (Frankham, 1995; Ralls & Ballou, 1986). Traditionally, the analysis and monitoring of inbreeding effects in captive colonies has been based on studbook data (Ralls & Ballou, 1986). However, gathering pedigree data accurately is not possible in most cases and assumptions made in the absence of data have been shown to influence inbreeding coefficient calculations (i.e., in three different endangered gazelle species, Ruiz‐López, Roldán, Espeso, Gomendio, 2009). Molecular genetic analyses do not require prior pedigree information for kinship estimation and can provide much more realistic insights into relationships within captive populations. Such analyses have recently led to important improvements to the genetic management of captive stock (Witzenberger & Hochkirch, 2011).

**Figure 1:**
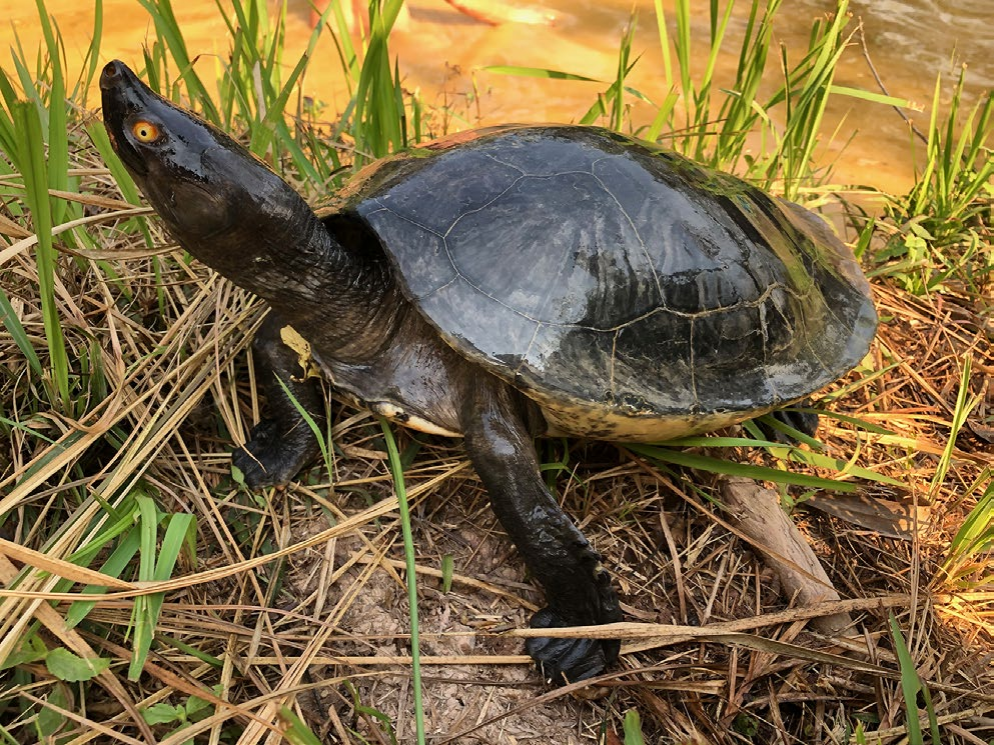
A juvenile *Batagur affinis edwardmolli* photographed by Brett Baldwin in The Koh Kong Reptile Conservation Center, Cambodia

The Southern River Terrapin, *Batagur affinis* Cantor, 1847, is a freshwater turtle species that once inhabited big rivers in Vietnam, Cambodia, Thailand, peninsular Malaysia, Singapore, and Sumatra (Moll et al., 2015). Currently it is listed as Critically Endangered by the 2000 IUCN Redlist (Horne, Chan, Platt, & Moll, 2016) and as one of the world's 25 most endangered freshwater turtles and tortoises by the Turtle Conservation Coalition (2011). Based on minor morphological differences, coloration, nesting ecology, as well as three mitochondrial (mtDNA) and three nuclear DNA markers, Praschag et al. (2009) divided *B. affinis* into two subspecies: the western nominate race, *B. affinis affinis*, and an eastern race *B. affinis edwardmolli* (Figure 1) (Moll et al., 2015). Today the nominate subspecies is only found on the western coast of the Malay Peninsula; it is probably no longer found in Sumatra (Mistar & Singleton, 2012). Conversely, the eastern subspecies *B. a. edwardmolli* used to extend from Singapore to Indochina but is now thought to be extinct in the wild at least in Singapore, Thailand, and Vietnam (Moll et al., 2015). In Cambodia, a small population was rediscovered in 2001 (Platt, Stuart, Sovannara, Kheng, & Kimchhay, 2003), and a juvenile was subsequently found in a district neighboring that of the rediscovery site (Holloway & Sovannara, 2004). Overall *B. a. edwardmolli* therefore persists in the form of very small populations on the east coast of the Malay Peninsula and the relict population found in Cambodia (Moll et al., 2015), making the Cambodian population the only remaining genetic stock across all of Indochina.

**Figure 2:**
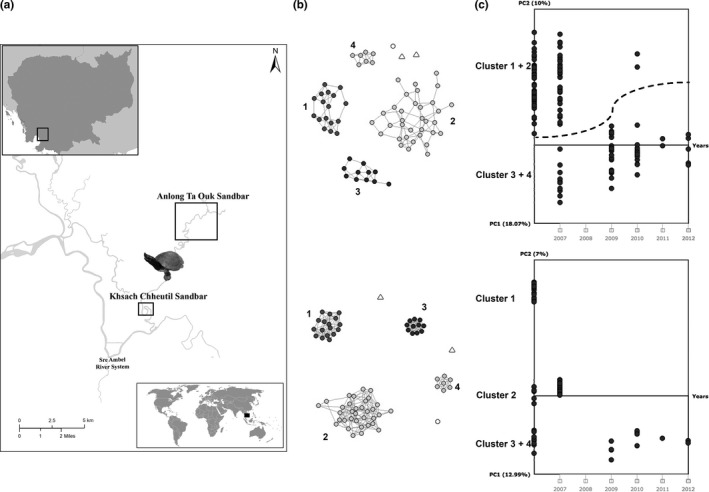
(a) Location of *Batagur affinis* nesting sites (squares) in the Sre Ambel River System, southern Cambodia. (b) The mutual *k*‐nearest group graph (SNP‐based network) for *k* = 5 (top) and *k* = 10 (bottom). Each shape represents an individual; different shapes indicate different 784 bp mtDNA haplotypes. Equal shape colors indicate full‐sibship except for ungrouped individuals. (c) Time series of microsatellite‐based (top) and SNP‐based PCoA (bottom) for 2006–2012

The remnant population of *B. a. edwardmolli* in Cambodia is known from Koh Kong province in the Sre Ambel River System (southern Cambodia) (Figure 2a). Conservation surveys over a 5 year period (2002–2007) demonstrated that the entire remnant population produced an average of 4.6 (range, 1–7) nests per year (Moll et al., 2015). The small numbers today reflect a combination of overharvesting and excessive egg collection for consumption, pollution, and the loss of habitat from sand mining (Iskandar & Erdelen, 2006; Moll, 1990; Moll & Moll, 2000; Platt et al., 2008; Sharma & Tisen, 2000). Since 2006, hatchlings have been collected from nesting sites along the river (Figure 2a) and kept at a sanctuary that is managed by the Wildlife Conservation Society and the Cambodian Fisheries Administration.

The turtle conservation program in Cambodia includes in‐situ and ex‐situ conservation measures such as fencing wild nests to protect hatchlings from potential predators, protecting remaining nesting sites along the Sre Ambel River, transferring new hatchlings to secure sites, and rearing of hatchlings in captivity (Moll et al., 2015). One of the goals of the conservation program of the Sre Ambel population is to establish assurance colonies given the extremely low number of nests per year and the ongoing threats to their survival.

We sampled 136 individuals in captivity, which equals ~30% of the remaining Indochinese population of *B. a edwardmolli,* given critically low numbers of individuals left in the wild. We utilized genome‐wide single nucleotide polymorphisms (SNPs) obtained via double digest restriction associated DNA sequencing (ddRAD‐seq) (Peterson, Weber, Kay, Fisher, & Hoekstra, 2012). Additionally, we amplified 10 microsatellite markers from 136 samples, and a fragment of 784 bp of mtDNA (including the partial ND3 gene and partial control region) from 50 samples. Our aims were (a) to estimate the number of breeders producing nests in the wild population from 2006–2012; (b) to infer an optimal set of potential breeders in captivity sired by wild breeders for maximizing genetic diversity and minimizing inbreeding in the assurance colony so that the deleterious and potentially rapid effects of inbreeding depression will be prevented in the future genetic stock of this relict population.

## 2 MATERIALS AND METHODS

### 2.1 Sampling and DNA extraction

During the nesting seasons in 2006–2012, from December to early March, a team consisting of six nest guards went for nest searches along the Sre Ambel River System for 15 days per month (S. Som, per. obs.). Nest searches were conducted in the early mornings to ensure female tracks on sand beaches had not been washed out. Once found, nests were fenced for protection from natural predators and guarded by the field guards. All hatchlings were health checked and transported to the wildlife sanctuary to form the captive population after emerging from within the fences. These individuals comprise the cohorts of 2006–2012, except for 2008 when no turtle nests were found. Every year only one nest was found on either of the corresponding beaches, except for 2006 when three nests were found (Table 1).

**Table 1:** Number of nests found between 2006–2012, their locations, number of hatchlings collected, their inferred parent sets, and network cluster assignments

Year	Location of the sandbar	No. of nests	No. of hatchlings collected	Parent sets (F: female, M: male)	Network cluster assignments
2006*	Anlong Ta Ouk	3	30	F1 × M1, F2 × M2, F3 × M3	1, 2, 4
2007	Khsach Chheutil	1	28	F2 × M2	2
	Anlong Ta Ouk	2			
2008	None	0	0	None	None
2009	Khsach Chheutil	1	4	F3 × M4	3
2010	Khsach Chheutil	1	4		3
	Anlong Ta Ouk	1			
2011	Khsach Chheutil	2	1		3
2012	Khsach Chheutil	1	2		3

* There is one ungrouped individual in the network cluster collected in 2006 from an unknown location along the Sre Ambel River. The parent set of this individual is not shown in the table.

We collected tissue samples from the rear‐foot webbing of all 136 turtles in the only captive Cambodian population as of 2012. In total, 134 of these individuals are known to have originated from the Anlong Ta Ouk and Khsach Chheutil sandbars in the Sre Ambel River System, southern Cambodia (Figure 2a), whereas the remaining two samples with an unknown origin had been contributed by fishermen.

A total of 102 individuals are of known sex, namely 66 males and 36 females. In handling the turtles, we followed the Guidelines on the Care and Use of Animals for Scientific Purposes provided by the Singapore National Advisory Committee for Laboratory Animal Research and the National University of Singapore Institutional Animal Care and Use Committee.

DNA was extracted from tissue samples following standard procedures using the DNeasy Blood and Tissue Kit (Qiagen Corporation). DNA concentrations were measured with a dsDNA High Sensitivity Assay Kit by Qubit 2.0 Fluorometer (Invitrogen).

### 2.2 Microsatellite genotyping and analysis

Ten tetra‐, one tri‐, and one pentanucleotide repeat loci, originally developed for *Batagur trivittata* (Batr4, 9, 10, 14, 16, 22, 25, 26, 30, 31, 39, 48; Love, Hagen, Horne, Jones, and Lance (2013)) were amplified for all the DNA samples (*n* = 136) following the protocol of O'Bryhim, Chong, Lance, Jones, and Roe (2012). All samples were genotyped using the ABI 3130 platform, and allele sizes were scored using GeneMapper v.4.0 (Applied Biosystems). All individuals were screened at all loci at least twice to confirm repeatability and accuracy in PCR amplification and fragment size scoring. To transform allele size into integers and avoid rounding errors, we normalized fragment scores by using TANDEM (Matschiner & Salzburger, 2009). For subsequent analysis, normalized allele sizes were used.

The presence of null alleles was checked with MICRO‐CHECKER (Van Oosterhout, Hutchinson, Wills, & Shipley, 2004). Tests for deviation from Hardy–Weinberg equilibrium and linkage disequilibrium for all pairs of loci were performed using GENEPOP'007 (Rousset, 2008). Lastly a test of neutrality was performed with BayeScan v.2.1 (Foll & Gaggiotti, 2008) with default settings.

### 2.3 ddRAD‐seq library preparation and sequencing

In addition to microsatellite genotyping, genome‐wide SNPs were mined by following a ddRAD‐seq approach (Peterson et al., 2012). In silico double digests using EcoRI‐MspI, EcoRI‐SbfI, EcoRI‐SphI, and SpfI‐SbfI were performed on the basis of the western painted turtle (*Chrysemys picta bellii*) genome as it is the phylogenetically closest available genome for *B. affinis*, using the SimRAD package within R (Lepais & Weir, 2014; R Core Team, 2015). In the end, samples that provided at least 200 ng of DNA (*n* = 129) were digested using the EcoRI and MspI enzymes, because this enzyme combination yielded a maximum number of in silico RAD loci with the target size selection window of 250–650 bp (Figure S1). A single ddRAD‐seq library was prepared by processing the 129 samples following the protocol by Chattopadhyay et al. (2016). The quality check of the final library fragment range was performed on a Fragment Analyzer Automated CE System (Advanced Analytical Technologies). Finally, 10 p.m. product from the quality checked library was sent to the Singapore Centre of Environmental Life Sciences Engineering in Nanyang Technological University, Singapore, and sequenced on an Illumina HiSeq 2500 platform for a paired end run in one lane, yielding read lengths of 150 bp.

### 2.4 Genome‐wide SNP generation and analysis

Data quality of the sequences was assessed using FastQC v0.11.3 (Andrews, 2010). Raw data were demultiplexed in STACKS v1.44 (Catchen, Hohenlohe, Bassham, Amores, & Cresko, 2013) using process_radtags. Sequences with a raw phred score below 20 were discarded. Reads with uncalled bases or low‐quality scores were removed, and reads were truncated to 140 bases to eliminate potential sequencing error occurring at the ends of reads.

De novo and reference‐based locus assemblies were conducted separately by running denovo_map.pl and ref_map.pl scripts within STACKS (Catchen et al., 2013), respectively. The former script constructs loci for each sample, whereas the latter one uses previously constructed loci from reference genome alignments. Both programs construct catalog loci and match individual loci to the catalog.

For de novo assembly only the forward read was analyzed and no more than one SNP per locus was admitted to preclude linkage bias. Minimum depth of coverage (m), minimum mismatches in creating individual stacks (M), and mismatches in secondary reads (*n*) were set to 7, 2, and 1, respectively. Highly repetitive RadTags were removed to avoid inclusion of paralogs. The resultant SNP set was used in subsequent analyses as SNP set A.

For reference‐based assembly, forward and reverse sequence reads from individuals were aligned to the western painted turtle (*C. picta bellii*) genome using the BWA‐MEM alignment algorithm (Li, 2013). Reads that did not properly pair in mapping or that had a phred score <20 were discarded. All alignments were sorted by leftmost coordinates using the SAMTOOLS software (Li et al., 2009). The ref_map.pl program of STACKS (Catchen et al., 2013) was run with minimum depth of coverage set to 7, and the resultant SNP set was used in subsequent analyses as SNP set B.

Once catalog loci and matches were identified with both denovo_map and ref_map approaches, the “populations” program in STACKS (Catchen et al., 2013) was run allowing for 20% missing data (r). PLINK v1.07 (Purcell et al., 2007) was used to remove individuals with >10% missing loci. For both sets A and B, three options for pruning SNPs with minor allele frequencies (MAF) <0.05, <0.01, 0 were applied, resulting in six final datasets (A1‐3, B1‐3). Neutrality tests were conducted for SNPs across all datasets using BayeScan v2.1 (Foll & Gaggiotti, 2008) under default settings. Also mean polymorphic information content (PIC) of the SNPs across all datasets was calculated with CERVUS 3.03 (Kalinowski, Taper, & Marshall, 2007). The dataset that provided the highest number of individuals with the highest mean PIC (SNP set A2, Table 2), including 72 individuals (40 males, 19 females, 13 unknown sex, see Table S1) with 2,658 SNPs (mean PIC = 0.28), was used for all subsequent analyses.

**Table 2:** Number of single nucleotide polymorphisms (SNPs) remaining after minor allele frequency (MAF) pruning, mean polymorphic information content (PIC) and number of remaining individuals across all six datasets produced

	No. of remaining individuals	No. of SNPs remaining after a pruning regime using…		
		MAF < 0.05 (1)	MAF < 0.01 (2)	MAF = 0 (3)
de novo approach (SNP set A)	72	1,809 (mean PIC = 0.21)	2,658 (mean PIC = 0.28)	3,459 (mean PIC = 0.26)
ref_map approach (SNP set B)	68	2,909 (mean PIC = 0.26)	3,410 (mean PIC = 0.22)	3,480 (mean PIC = 0.18)

### 2.5 Population structure

Our dataset was expected to violate one of the major assumptions of model‐based population structuring: unrelatedness (see Anderson and Dunham (2008) and Rodriguez‐Ramilo and Wang (2012)), because a small number of individuals remains in the wild, many of which are potentially closely related. Therefore, population structure was analyzed via Principal Coordinate Analysis (PCoA) (Gower, 2015) as a multivariate method designed for clustering related individuals. Pairwise PhiPT distance matrices were generated for both microsatellite and SNP datasets separately, and the patterns of genetic relationship within these matrices were visualized with PCoA as implemented in GenAlEx v.6.5 (Peakall & Smouse, 2006, 2012). To visualize how genetic structure of the samples has changed through the sampling years, the first two principal components (PCs) of the SNP‐ and microsatellite‐based PCoAs were plotted against the sampling years with 3D Scatter Plot v2.1 (Doka, 2013) macro in Microsoft Excel (2011). The time series plots were rotated along the three axes so that all individuals are aligned perpendicularly across sampling years. Additionally, a network‐based approach was performed for the SNP dataset by using the R package “Netview” (Neuditschko, Khatkar, & Raadsma, 2012; R Core Team, 2015; Steinig, Neuditschko, Khatkar, Raadsma, & Zenger, 2016), as it provides fine‐scale population structure resolution. Initially, an NxN identity by state pairwise distance matrix was generated in PLINK (Purcell et al., 2007). The Netview pipeline utilizes the distance matrix to construct mutual k‐nearest neighbor graphs (mkNNGs). To choose an appropriate *k* for the mkNNG, community detection algorithms of Fast‐Greedy, Infomap, and Walktrap were performed. The resulting number of clusters *n* was plotted against *k*. When the selected algorithms showed a general congruence with some variation in individual resolution across the mkNNGs, the lowest *k* value was selected to visualize the genetic network, as low values of *k* are expected to detect a large number of clusters (Neuditschko et al., 2012).

### 2.6 Population genetic diversity and assurance colony management with SNP data

Observed heterozygosity (*H*_o_), expected heterozygosity (*H*_e_, gene diversity (Nei, 1987)), and inbreeding coefficients (*G*_IT_, relates *H*_o_ to the expected heterozygosity within populations, *H*_s_ (Nei, 1987)) were calculated with GenoDive v2.0b23 (Meirmans & Van Tienderen, 2004). Global tests of heterozygote excess and deficiency were performed to test for deviation from Hardy–Weinberg equilibrium in GENEPOP version 4.2 (Rousset, 2008). Inbreeding coefficients for each individual (Ritland, 2009) and pairwise relatedness (PR) (Lynch & Ritland, 1999) as well as internal relatedness (IR) (a measure of individual heterozygosity (Amos et al., 2001)) were estimated using COANCESTRY v1.0.1.7 (Wang, 2011), the R package Related (Pew, Muir, Wang, & Frasier, 2015) and RHH (Alho, VäLimäki, & Merilä, 2010), respectively.

Our assurance colony management strategy depends on minimizing the average PR across the group and IR of individuals included in the group. Therefore, we ran SWINGER (Sandoval‐Castillo et al., 2017), utilizing the IR table incorporating sex information of the individuals and the PR table. The threshold values for maximum IR and maximum PR were both set to average values of the data. Maximum average PR was set to within 90% of maximum PR. Then the program was run so as to return 12 females and 13 males appropriate for this setting. A 10% decrease for all values was applied for each subsequent run until parameters became too stringent to provide any more results.

### 2.7 Sibship assignment and parental genotype reconstruction with SNP data

Sibling analysis and pedigree genotype reconstruction were performed using a nearly exhaustive run of the Full‐Likelihood method in COLONY (Jones & Wang, 2010) with female and male polygamy allowed. A range of genotyping error rates was tested with 10%, 1%, and 0.1% across loci. The non‐inbreeding model was selected and a sibship prior was not included. Parentage was accepted when the three error rate runs provided the same parent dyads with posterior probability values >90%. Inferred loci with three genotyping error rate runs were included in the parental genotypes when assigned likelihoods >0.90 (three distinct datasets for each parent). Loci with missing data and MAF >0.01 were removed by PLINK (Purcell et al., 2007). To understand if the relatedness of the parent sets affects the number of genetic clusters obtained by SNP‐ and microsatellite‐based data, average relatedness (Lynch & Ritland, 1999) between parent sets of each genetic cluster was calculated with COANCESTRY (Wang, 2011). Specifically, we tested if PR between parent set combinations is significantly different from overall average relatedness of parent sets by performing 10,000 bootstraps in COANCESTRY (Wang, 2011).

To test if assigned full‐sib families could have resulted from chance alone, the relatedness among all samples in a family group was calculated using the Wang estimator (Wang, 2002) in the R package Related (Pew et al., 2015). Samples within each group were then randomly shuffled 1,000 times using the grouprel function in Related and average expected relatedness values were compared with observed ones. The package was also utilized for the calculation of PR between inferred parental genotypes.

### 2.8 MtDNA analyses

Fifty samples were randomly selected from the genetic clusters obtained from the above‐mentioned population structure analyses. Given that ddRAD‐seq libraries contained numerous mtDNA sequences of the samples that were cut by EcoRI and MspI, de‐multiplexed and cleaned individual sequences of these 50 samples were aligned against the full mtDNA genome of *Batagur trivittata* (GenBank accession: NC_032300), the phylogenetically closest species for which an mtDNA genome is available, by using CLC GENOMICS WORKBENCH 10 (Qiagen). For each individual, aligned sequences >20× coverage were retained. Recovered regions were located between positions 4,399–4,544 in the mtDNA genome, encompassing 145 bp from the ND3 gene.

Additionally, a primer set of 5′‐TTTTTCCCCTAGCATATCACCA‐3′ (forward) and 5′‐AGTTGCTCTCGGATTTAGGG‐3 (reverse) was manually designed for the amplification of a 639 bp fragment of the mtDNA control region using the software PRIMER3 (Koressaar & Remm, 2007; Untergasser et al., 2012). PCR reactions were prepared with 20 ng of genomic DNA, 10 µM of each primer, 1X Q5 Reaction, 0.02 U/µl Q5 Hot Start High‐Fidelity DNA Polymerase and nuclease‐free water to top up the mix to a total volume of 25 µl. Thermocycling conditions for the PCR involved initial denaturation at 98°C for 30 s, followed by 35 cycles of 98°C for 10 s, 64°C for 30 s, and 72°C for 30 s, with a final 2‐min extension at 72°C. Finally, PCR products were purified with the Gene Mark Gel Extraction Kit (Hopegen Biotechnology). Sequencing reactions were performed with an ABI 3730XL kit (Applied Biosystems) at Mclab. PCR products were sequenced in both directions to increase accuracy. MtDNA sequences representing distinct haplotypes were deposited in GenBank under the accession numbers MN069309 and MN069310. The alignment of concatenated mtDNA sequences (partial ND3 gene and partial control region) was performed using the CLUSTALW algorithm in MEGA 7 (Kumar, Stecher, & Tamura, 2016). Nucleotide differences between haplotypes were calculated by dividing total number of nucleotides with the number of nucleotides showing difference.

## 3 RESULTS

### 3.1 Microsatellite analyses

In total, 12 loci from 136 individuals were genotyped with overall 0.14% missing data. The mean number of alleles per locus was 5.20. Two loci (Batr 25 & 30) were excluded from subsequent analyses as they showed significant levels of null alleles. Six of 10 loci showed deviations from Hardy–Weinberg equilibrium; of these six loci, five showed a significant excess in heterozygotes. Linkage disequilibrium was observed for 23 pairs of loci across the entire dataset, corresponding to the 52% of all pairs. These results are most likely due to the small population size and sampling of sibling groups, not actual physical linkage of sites. None of the loci significantly deviated from neutrality. Therefore, all 10 microsatellite loci were used for the population structure analysis.

### 3.2 Genome‐wide SNP generation and analyses

Samples from 129 individual turtles yielded a total of 598,211,204 bp of raw data. Six of these samples were immediately removed (5% of the total number of individuals), corresponding to <0.03% of all reads, due to poor data recovery (<250 kb). Eventually, 72 individual samples in SNP set A and 68 samples in SNP set B remained after individuals having >10% missing loci were eliminated.

De novo and reference‐based mapping of loci, followed by missing data and MAF filtering, yielded six different SNP datasets (Table 2). We conducted downstream analyses using SNP set A2 (2,658 SNPs, mean PIC = 0.28; Table 2) because it includes SNPs from the highest number of individuals with the highest mean PIC value (Çilingir et al., 2017).

### 3.3 Population structure

The PCoA performed with 10 microsatellite loci provided two loose genetic clusters (Figure S2), whereas the one performed with 2,658 genome‐wide SNPs yielded three distinct clusters and three individual samples that did not group into any of the clusters (Figure S3). The network analysis resulted in groupings of four distinct clusters and three ungrouped individuals instead (Figure 2b, see Table S1).

Cluster 1 and 2 of SNP‐based network analysis matched with cluster 1 and 2 of the SNP‐based PCoA (Figure S3), respectively; whereas cluster 3 of the SNP‐based PCoA (Figure S3) comprises the individuals from clusters 3 and 4 of the network analysis (Figure 2b,c). Cluster 1 and 2 of microsatellite‐based PCoA (Figure S2) matched with clusters 1–2 and 3–4 in network analysis, respectively (Figure 2b). This outcome shows that microsatellite‐based population structure results are congruent with SNP‐based ones, but that the latter provide a finer resolution in clustering.

The first two PCs of the SNP‐ and microsatellite‐based PCoAs were plotted against sampling years. The time series of both types of data show a massive decline in population structure even within the short time frame of 7 years (2006–2012) (Figure 2c).

### 3.4 Population genetic diversity and assurance colony management using SNP‐based data

Based on our genome‐wide SNPs, we calculated an overall observed heterozygosity of *H*_o_ = 0.213 and a slightly lower expected heterozygosity of *H*_e_ = 0.208, but the null hypothesis of heterozygote excess was rejected (Score [*U*] test (Rousset & Raymond, 1995), *p* = 0.0001). The inbreeding coefficient *G*_IT_ was calculated as −0.025 ± 0.005. Individual inbreeding coefficients ranged from −0.225 to 0.27 (Table S1). The average PR among 59 previously sexed individuals was estimated as −0.015. The mean IR was estimated as −0.61 with a range of −0.43 to 0.23 (Table S1). Following the parameter selection methodology of Sandoval‐Castillo et al. (2017) we obtained one list of 12 females and 13 males with an average PR of −0.075 and a mean IR of −0.45.

### 3.5 Sibship assignment and parental genotype reconstruction with SNP data

Parent sets were successfully assigned for 95.9% of individuals via sibship assignment analysis with COLONY (Jones & Wang, 2010). The three ungrouped samples in the genetic network had <0.9 posterior probability assignments, and were therefore excluded from further family assignment (ungrouped individuals in Figure 2b). A total of four full‐sib families were identified, two of which shared one parent (half‐sib). Each network cluster provided one full‐sib family (Figure 2b), while SNP‐based PCoA (Figure S3) grouped the two half‐sib families together.

By permuting the sample dataset, we found that the average relatedness within each full‐sib group is significantly higher (*p* < 0.001) than a random distribution of relatedness values (Figure S4).

Given that four full‐sib families were identified, two of which shared one parent, a total of seven parental genotypes were reconstructed with COLONY (Jones & Wang, 2010). Despite having no nest location data, because every year except 2006 there was one nest collected from the nesting beaches (one mother for each cohort), we estimated that the current captive individuals were sired from four male and three female breeders. For these seven breeders, allelic states for 1,809 out of 2,658 loci were inferred with assigned likelihoods of >0.9. Genotyping error rates of 10%, 1%, and 0.1% were tested across loci followed by missing data and MAF filtering, resulting in 88, 126, and 146 SNPs among parent sets, respectively. For all three datasets, the average relatedness between parent sets of clusters 1 and 2 (*n* = 2 + 2 = 4) was insignificantly higher than the average relatedness across all parent sets, whereas the average relatedness between parent sets of clusters 3 and 4 (*n* = 3, because they share one parent) was shown to be significantly lower than across all sets at a 95% confidence level (Table 3).

**Table 3:** The assigned parent sets of the network analysis clusters, their average relatedness values within each set and among the other parent sets that were calculated with three different genotyping error rates are shown

Genetic cluster #	Parent sets	Genotyping error rates					
		10%		1%		0.01%	
		Average r within the parent set	Average r among the other parent sets	Within	Among	Within	Among
1	P1 × P2	0.4	0.032 ± 0.01	0.3	0.03 ± 0.01	0.3	0.03 ± 0.01
2	P3 × P4	0.4	0.036 ± 0.01	0.4	0.03 ± 0.01	0.4	0.03 ± 0.01
3	P5 × P6	−1.4*	0.037 ± 0.001	−1.3*	0.032 ± 0.001	−1.3*	0.033 ± 0.001
4	P5 × P7	−0.4*	0.036 ± 0.001	−0.9*	0.03 ± 0.001	−0.9*	0.03 ± 0.001

* Statistical difference (*p* < 0.05).

### 3.6 MtDNA sequencing

A total of 784 bp of mtDNA sequence yielded only one haplotype across all four groups and one ungrouped individual (white circle in Figure 2b). The other two ungrouped individuals (white triangles in Figure 2b) shared an alternative mtDNA haplotype that differed in two base pairs from the major haplotype, equal to 0.02% sequence divergence (GenBank accession numbers: MN069309 and MN069310).

## 4 DISCUSSION

### 4.1 Performance of the different marker sets used in this study

We found four genetic clusters in our dataset spanning 72 individuals of the sole remnant Indochinese population of *B. a. edwardmolli*. Our analyses suggest that each of these four genetic clusters (Figure 2b) comprises one full‐sib family, two of which shared the same mother (as there is only one nest found for each cohort represented in the half‐sib families). SNP‐based PCoA revealed only three genetic clusters (Figure S3; Figure 2c), combining the two half‐sib families into one cluster, whereas microsatellite‐based PCoA yielded two groups comprising the genetically more closely related parents' offspring clustered together (Figure S2; Table 3; Figure 2c). Not surprisingly >2,000 genome‐wide SNPs outperformed 10 microsatellite loci in providing finer resolution of population structure, mirroring similar cases in Atlantic peacock wrasse (Carreras et al., 2017), sperm whales (Mesnick et al., 2011), and Tasmanian devils (Hendricks et al., 2017). Our study was not directly designed for the comparison of the power of genome‐wide neutral SNPs and microsatellites on parentage and relatedness analyses. Recent studies aimed to do so have shown that even few hundreds of genome‐wide SNPs outperform microsatellites in parentage assignment accuracy and relatedness estimation (Flanagan & Jones, 2019; Thrasher, Butcher, Campagna, Webster, Lovette, 2018), including in very small populations (Andrews et al., 2018).

### 4.2 Catastrophic drop in population size and diversity of Cambodian *B. affinis* in the wild

Based on our estimates eight wild breeders were present in 2006 (three females, three males based on parentage analyses, and two potential parents from an ungrouped individual hatched in 2006), followed by a drop to two in 2007, and to zero (no nests were found) in 2008 (Table 1). Our pedigree analysis showed that between 2009 and 2012, a female breeder from 2006 started breeding with a male breeder, which is potentially newly started contributing to the gene pool (Table 1). In addition to declining numbers of breeders, the number of hatchlings collected dropped from 31 to 1–4 each year after 2007. The main reason of this drop is likely a commercial license for sand mining along the Sre Ambel River given to a private company from 2007 to 2017, which eventually destroyed nesting beaches along the river (B. Horne, personal observations). All in all, we demonstrated that, only within 7 years, the Cambodian *B. affinis* population in the wild has undergone a massive decrease in population structure (Figure 2c) that highlights the extreme urgency for ex‐situ measures to help conserve this demographically and genetically impoverished population.

### 4.3 Valuable breeders for the assurance colony

Our sample set mostly includes half‐ and full‐sibs that were sired by <10 breeders in the wild. Consequently, any unrelated individuals to these families will be of highest importance for representing the remnant wild genetic variation within the captive population. Therefore, three of the ungrouped individuals (two males, one female) that were merged with the main clusters of microsatellite‐based analyses but represented in SNP‐based clustering analyses were assigned as valuable breeders for the assurance colony. One of these males belongs to the earliest (2006) cohort from the Sre Ambel population (S. Som, personal observations) and it shares the same 784 bp mitochondrial haplotype with other individuals hatched in Sre Ambel. This individual might be the only surviving hatchling from a single clutch belonging to an unknown location in the Sre Ambel River System. The remaining ungrouped male and the female have an unknown origin and had been labelled as Big Boy and Big Mama, respectively. Big Mama was thought to belong to a Cambodian population of unspecified provenance, and Big Boy was thought to hail from the Sre Ambel population (B. Horne, personal observations). Given that Big Mama and Big Boy share the same 784 bp mitochondrial haplotype that is 0.2% different from the remaining Sre Ambel population, we assume that these two individuals might belong to another maternal lineage in the expanded, pre‐bottlenecked Sre Ambel population. The existence of these unrelated individuals will boost genetic diversity in a self‐sustaining assurance colony.

### 4.4 Applied conservation genomics of *B. affinis*

Southern river terrapins were reportedly common in the Mekong drainage including the Tonle Sap lake system until the late 19th century (Moll et al., 2015). They have been widely extirpated since, and only one known *B. affinis* population currently remains in Cambodia (Platt et al., 2008, 2003). Given extensive efforts to collect all wild hatchlings from 2006–2012, we assume that the genetic diversity and structure uncovered in this study directly reflects actual genetic diversity of the entire remaining Indochinese population of this species.

As previously observed in *Batagur trivittata* (Çilingir et al., 2017), the sister species of *B. affinis*, global *G*_IT_ is smaller than zero (global *G*_IT_ = −0.025 ± 0.005; individual inbreeding coefficients −0.225 to 0.27; Table S1). This could be interpreted as a lack of severe inbreeding, assuming our dataset represents only the first generation of a potentially long‐lived turtle's population (estimated age of maturity is 25 years; Moll et al., 2015) whose bottleneck is relatively recent. However, marker‐based inbreeding estimators rely on multiple assumptions and vary with the demography of populations, and our sample set is likely to violate some of these assumptions. For example, when parental allele frequencies are unknown, population allele frequencies are calculated based on the current sample (in our case the first generation offspring of wild breeders), and when the current sample contains highly related individuals (our sample set comprises many half‐ and full‐sibs) it has been shown that the marker‐based inbreeding estimators may return misleading results smaller than zero (Wang, 2014). Moreover, the population in Cambodia has an extremely small effective population size, another potential source of bias that may lead to *F*_IS_‐related inbreeding coefficients below zero (Waples, 2014). Ultimately, given the severity of the bottleneck and the strong kinship divisions in the population, we assume the presence of inbreeding but our *F*_IS_‐related metric was probably unable to detect this. Ex‐situ conservation measures to minimize inbreeding within the assurance colony of Cambodian *B. affinis* are thus extremely important for the conservation of the species as inbreeding depressions can have immediate and exponentially growing effects on such small populations (Frankham, 2005; Reed & Frankham, 2003).

Retaining contemporary genetic diversity of the Cambodian population should be the major management focus, given the population is highly genetically and demographically impoverished. Accordingly, a fine‐scale assessment of population structure and pedigree analysis plays a key role in selecting ideal individuals for assurance colony management and/or reintroduction efforts. To this end, we generated a list of 25 individuals for a captive assurance colony—directly selected based on our population‐genomic data to maximize genetic diversity—which will be a suitable basis for future genetic stock for the species in Cambodia once the individuals have started breeding. Additionally, this methodology can be applied for the management of future reintroduction events, and our findings on population genetic inferences can be used as a baseline for future monitoring of the assurance and reintroduction colonies.

The need for genomic methods in the conservation of non‐model and non‐commercial species is growing (Shafer et al., 2016) despite an overall increase of conservation genomic studies (see examples in Shafer et al. (2015) and Garner et al. (2016)). We believe that our work is one of the case studies filling this scientific gap by shedding light on genomic variation, population structure, and demographic parameters regarding a relict population of one of the most critically endangered turtle species.

The majority of the world's turtles are threatened with extinction and among these *B. affinis* is in a particularly critical state. We showed that the small remnant wild population in Cambodia has decreased even more across the short span of this study. From 2006 to 2008, the number of active breeders dropped from eight to zero and from 2009 to 2012 only two active breeders were revealed. Accordingly, although our dataset comprised genetically highly related individuals (full‐sibs and half‐sibs), the genome‐wide analysis provided a fine resolution in determining population structure, whereas traditional genetic markers failed to do so.

We applied genomic methods to inform choices for assurance colony management, and to help preserve the genetic diversity of *B. a. edwardmolli* while bridging the gap between in‐situ and ex‐situ conservation. Given that ~70% of the world's most threatened turtles inhabit Asia, with comparable demographic histories to *B. affinis*, our genomic strategy has the potential to be applied to many other species in the mid‐term future.

## CONFLICT OF INTEREST

We do not have any conflict of interests.

## AUTHOR CONTRIBUTIONS

FGÇ, DPB and FER designed the study. BDH and SS assisted in tissue sampling, logistics and fieldwork. AS conducted microsatellite genotyping and analyses under the assistance of FGÇ. FGÇ performed NGS library preparation and conducted bioinformatic analyses. FER supervised molecular work and bioinformatic analyses. FGÇ wrote the first draft with extensive input from FER, and all remaining authors contributed to the review of the manuscript.

## Data Availability

MtDNA haplotypes used in this study were deposited in the NCBI Nucleotide Database under accession numbers MN069309 and MN069310. The raw ddRAD sequences of all the individuals were deposited in the NCBI Sequence Read Archive under BioProject accession number PRJNA541109.
