# Supplementary material for: Last exit before the brink: Conservation genomics of the Cambodian population of the critically endangered southern river terrapin
Source: Ecol Evol. 2019 Aug 5;9(17):9500–10. doi: 10.1002/ece3.5434 (PMC6745661; doi:10.1002/ece3.5434)
Supplement: Supplementary file 1 [file ECE3-9-9500-s001.docx]

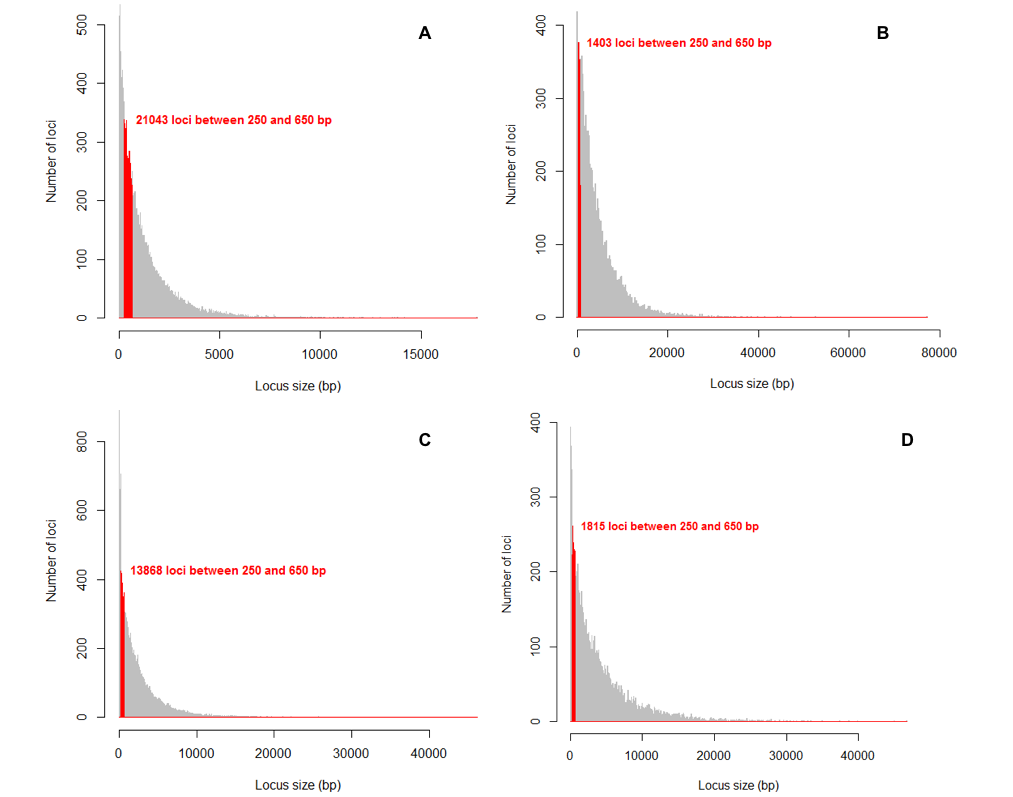


**Fig. 1** *In silico* double digests of *C. picta bellii* with A) EcoRI and MspI B) EcoRI and SbfI, C) EcoRI and SphI, and D) SpfI and SbfI with the selection window of 250-650bp is shown.


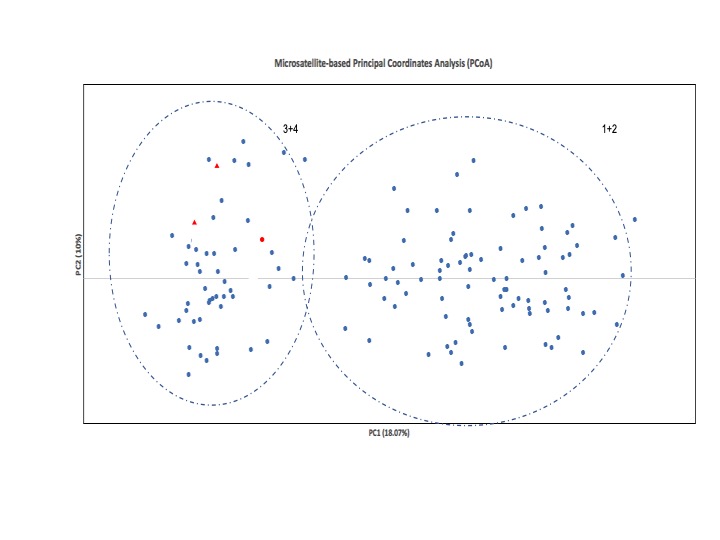


**Fig. 2** The microsatellite-based PCoA revealed two loose clusters. Each shape represents an individual; different shapes indicate different 784bp mtDNA haplotypes. Red color represents the ungrouped individuals of the network analysis (Fig. 2B). The clusters were numbered according to the cluster numbers of the network analysis (Fig. 2B).


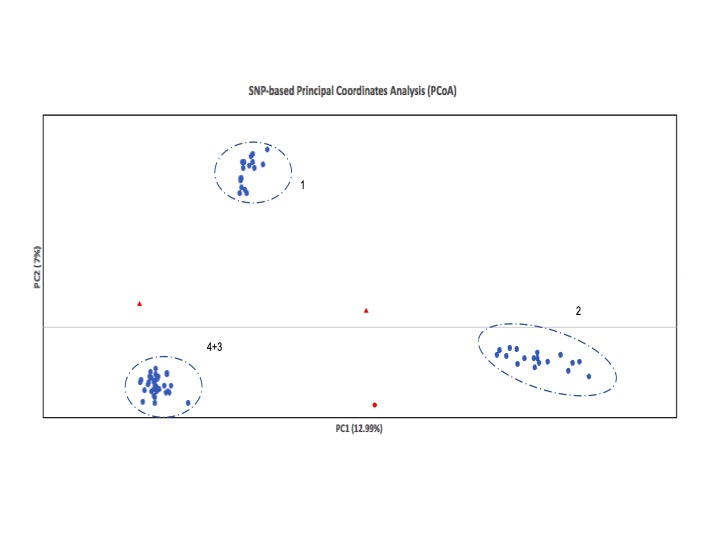


**Fig. 3** The SNP-based PCoA revealed three clusters. Each shape represents an individual; different shapes indicate different 784bp mtDNA haplotypes. Red color represents the ungrouped individuals of the network analysis (Fig. 2B). The clusters were numbered according to the cluster numbers of the network analysis (Fig. 2B).


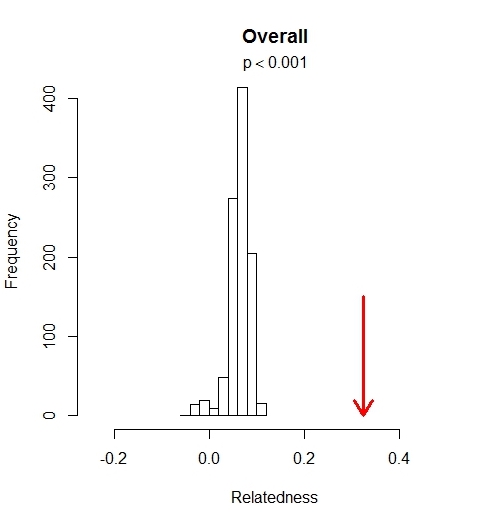


**Fig. 4** Expected relatedness values within each full-sib and half-sib families revealed with the pedigree analysis are shown. Red arrow represents the observed relatedness.
